# Identifying Care-Related Needs of Family Caregivers of People With Dementia: Beyond the Stress Process Model

**DOI:** 10.1093/geroni/igaa057.241

**Published:** 2020-12-16

**Authors:** Sara Moss, Lauren Gebhardt-Kram, Holly Dabelko-Schoeny, Jennifer Cheavens

**Affiliations:** 1 Ohio State University, Columbus, Ohio, United States; 2 The Ohio State University, Columbus, Ohio, United States

## Abstract

The psychosocial stress process model (Pearlin et al., 1990) remains a dominant theoretical framework characterizing the transactions between factors affecting outcomes of informal caregivers of people with dementia (PWD). Despite widespread agreement that the model provides an important framework for understanding caregiver experiences and predicting caregiver outcomes, it is not sufficient to clarify the needs of caregivers. Needs are conceptualized as the skills and resources that could be used to ameliorate the negative impacts of caregiving and promote quality of life (Gitlin & Hodgson, 2015). Determining caregiver needs requires appreciation of the complex background, contextual, and stress-related variables described in the stress process model and requires empirically- and theoretically-driven understanding of the diverse resources, materials, and skills that individuals require for global health and functioning. In this study, we conducted a content analysis of existing measures of dementia caregiver needs (N = 54), content analysis of materials related to evidence-based dementia caregiver interventions and government reports and documents (N = 28), and semi-structured in-depth interviews with current caregivers of PWD (N = 12) to identify the personal and care-related needs of family caregivers of PWD. We propose a framework of five inter-related need categories (Health-related needs, environmental needs, psychological needs, social needs, and needs related to the care and functioning of the PWD) that transact with the factors described in the stress process model, ultimately influencing functioning. In the future, we plan to test this model empirically with a nationally representative sample of caregivers.

